# Linkage mapping of total cholesterol level in a young cohort via nonparametric regression

**DOI:** 10.1186/1471-2156-4-S1-S92

**Published:** 2003-12-31

**Authors:** Saurabh Ghosh, Sarah Bertelsen, Theodore Reich

**Affiliations:** 1 Department of Psychiatry, Washington University School of Medicine, St. Louis, Missouri, USA; 2 Applied Statistics Unit, Indian Statistical Institute, Kolkata, India

## Abstract

**Background:**

Compared to model-based approaches, nonparametric methods for quantitative trait loci mapping are more robust to deviations in distributional assumptions. In this study, we modify a nonparametric regression method and the "contrast function"- based regression method to analyze total cholesterol level in the younger cohort (the offspring generation) of the Genetic Analysis Workshop 13 simulated data set.

**Results:**

We obtained significant evidence of linkage near four of the six non-sex-specific genes in at least 30% of the replicates.

**Conclusions:**

The proposed nonparametric method seems to be a powerful robust alternative to distribution-based methods.

## Background

Unlike qualitative or binary traits, which can be characterized completely by allele frequencies and genotypic penetrances, quantitative traits require an additional level of modeling: the probability distribution of the underlying trait. Thus, compared with model-based approaches like variance components, nonparametric methods for quantitative trait loci (QTL) mapping are more robust to deviations in distributional assumptions. Ghosh and Majumder [1] developed a nonparametric regression method based on kernel-smoothing for linkage mapping of QTLs using independent sib pairs. To analyze larger sibships, Ghosh and Reich [2] proposed a so-called "contrast function" that integrates trait values within a sibship into a linear combination whose coefficients sum to zero. Their test for linkage is based on a linear regression of the squared contrast function on a quadratic function of the estimated identity-by-descent (IBD) scores of all possible sib pairs within a sibship. As in the classical Haseman-Elston regression procedure and its extensions, the linear regression score decreases with increasing dominance at the trait locus. In this study, we propose a nonparametric regression method on the lines of Ghosh and Majumder [1] using the contrast function to perform a genome-wide scan of total cholesterol levels in the offspring cohort of the Genetic Analysis Workshop 13 (GAW13) simulated data set.

### Data description

For our analysis, we used longitudinal data (over five time points) on total cholesterol level and genome-wide information on 400 marker loci distributed over the 22 autosomal chromosomes for the offspring cohort. Our method utilizes cholesterol and marker data on 324 independent sibships (i.e., no two sibships considered are first degree relatives) of sizes varying from two to nine and their parental genotypes for IBD computations. We analyzed data on all 100 available replicates.

### Statistical methodology

Suppose *y*_*ijt *_denotes the total cholesterol level of the *j*^th ^sib in the *i*^th ^sibship at time point *t*, *i *= 1,2,...,324; *j *= 1,2,...,*n*_*i*_; *t *= 1,2,...,5; and  denotes the estimated IBD score for sibs *j *and *k *in sibship *i *at an arbitrary point *p *on the genome. Let **Y**_*i *_= ((*y*_*ijt*_)) be a *n*_*i *_× 5 matrix. Following Ghosh and Reich [2], we define, for the *i*^th ^sibship, a so-called contrast vector **c**_*i *_such that *c*'_i _**1 **= 0 and a square matrix  of order *n*_*i *_with the diagonal elements fixed at 0. For a fixed time point *t *(i.e., when *Y*_*i *_is a vector), Ghosh and Reich [2] developed a linear regression of the squared contrast function  on  to test for linkage at point *p *on the genome. For sibships of varying sizes, one needs to standardize both of these variables by a factor . In our longitudinal set-up, we also need to standardize the total cholesterol values over the five time points. Suppose the dispersion matrix of total cholesterol values over the five time points is estimated by , the 5 × 5 sample dispersion matrix computed using one sibling per sibship. Then, we define a modified contrast function as  and a corresponding quadratic function of the matrix of IBD scores . Based on statistical considerations [2], we propose the choice of , where the coefficient 1 is assigned at random to one of the sibs in the *i*^th ^sibship.

As pointed out in the Background, a linear regression of *U*_*i *_values on *W*_*i *_values deteriorates (i.e., the squared multiple correlation coefficient *R*^2 ^decreases) with increase in dominance at the QTL [2]. Thus, a more robust strategy is to estimate empirically the nature of the functional relationship between the two variables.

Following Ghosh and Majumder [1], we assume a nonparametric regression model:

*U*_*i *_= P(*W*_*ip*_) + *e*_*i*_; *i*=1,2,...,324,

where P is a real valued function and *e*_*i *_values are random errors. The functional form of P is estimated using a kernel smoothing technique [3] with kernel function:



The predictor of *U*_*i *_is given by:



where *h *is the "optimal" window length in the kernel smoothing procedure.

To assess the significance of our regression, we use a diagnostic measure [4]. One has to use resampling techniques such as bootstrap to obtain empirical thresholds under the null hypothesis of no linkage.

## Results

All of our analyses were performed prior to GAW13. The total cholesterol levels were corrected for age and sex using weighted least-squares linear regression. The IBD computations were performed using the statistical software MERLIN [5]. Since the number of alleles (38) at marker GATA21A06 on chromosome 9 exceeded the maximum allele capability of MERLIN, we discarded data on that marker from our analysis. We then performed the nonparametric regression of the contrast function on the quadratic function of the IBD matrix discussed above at every centimorgan on all 22 autosomal chromosomes. We set a *p*-value threshold of < 0.0001 (based on 10,000 bootstrap replications) to consider a linkage finding to be statistically significant. Since the "answers" were available to us, we considered a linkage peak to be true positive if it is within a 20-cM window (10 cM on either side) of the true position of a QTL. Hence, we have assessed the empirical power of detecting a QTL and the false-positive error rate of our nonparametric regression method based on the proportion of replicates yielding significant linkage peaks. The true positive linkage findings with empirical power > 0.3 and the false-positive peaks with error rate > 0.1 are presented below in Table 1.

We note that the non-sex-specific genes for total cholesterol level: b31, s7, b30, and b32 are located within the intervals of chromosomes 1, 7, 11, and 15, respectively, where we obtained significant evidence of linkage to an unobserved QTL in more than 30% of the replicates. We also found significant linkage near two other non sex-specific genes: b33 and s8 on chromosomes 3 and 15, respectively, but in less than 10% of the replications. Linkage near the sex-specific gene s9 on chromosome 21 was not significant in any of our replications. The false positive peak on chromosome 9 is within the interval containing b12, the major gene for HDL. It is possible that since total cholesterol level is highly correlated with HDL, the major gene for HDL showed significant linkage with total cholesterol level. There was no other region that yielded a false-positive rate greater than 0.05.

## Conclusions

Our proposed nonparametric method was able to detect linkage near four of the six non-sex-specific genes for total cholesterol level in multiple replicates. As expected, the rate of detection of the baseline genes increased with larger effects of the gene. We note that Martin et al. [6], analyzing total cholesterol level in the Framingham data set, also obtained evidence of linkage on chromosome 7 using the variance-components approach implemented in SOLAR. We also found that there was only one false-positive peak, which replicated in more than 5% of the replicates.

Since the proposed Δ statistic does not consider the direction of the relationship between the modified contrast function and the quadratic function of the matrix of IBD scores, there may be concern of an inflated false-positive error rate due to a random negative relationship between the variables under the null hypothesis of no linkage. To circumvent this problem, we ensured that the rank correlation between the variables was positive for each region showing significant evidence of linkage.

Currently used methods use LOD scores as a diagnostic to evaluate the significance of linkage peaks. Since our proposed rank correlation and kernel smoothing methods are nonparametric, a direct comparison with likelihood-based LOD scores is not possible. However, if we consider the *p*-values of our linkage peaks, we can theoretically obtain the LOD scores that would yield these *p*-values. For example, a *p*-value < 0.0001 can be attained for a LOD score greater than 3.29, while a *p*-value < 0.001 can be attained for a LOD score greater than 2.35. We are currently carrying out extensive simulations to compare the performance of the proposed procedure with existing model-based methods. Our preliminary comparisons with the regression procedure of Elston et al. [7] show that while their method has slightly higher power in the absence of dominance at the trait locus (in which case the linear regression is theoretically valid), the nonparametric regression procedure outperforms the linear regression procedure as dominance increases (Ghosh S, Majumder PP, Reich T, unpublished observations).

We finally emphasize that a major advantage of our method is that it does not assume any probability distribution for total cholesterol level or any specific functional form of dependence between the regression variables and is thus robust to violations in underlying model assumptions.

## Figures and Tables

**Table 1:** Linkage findings, genes, power, and false-positive rates.

**Chromosome**	**Interval**	**Gene**	**VE**^A^	**R**^B^	**Empirical Power**	**False-Positive Rate**
1	165–180 cM	b31	0.15	62	0.62	-
7	140–152 cM	s7	0.36	48	0.48	-
11	57–75 cM	b30	0.2	72	0.72	-
15	115–133 cM	b32	0.1	34	0.34	-
9	7–18 cM	-	-	12	-	0.12

^A^VE, variance explained by gene. ^B^R, replicates giving positive findings.
